# Towards shifted position-diffuse reflectance imaging of anatomically correctly scaled human microvasculature

**DOI:** 10.1038/s41598-020-74447-5

**Published:** 2020-10-15

**Authors:** Moritz Späth, Martin Hohmann, Clemens Roider, Benjamin Lengenfelder, Florian Stelzle, Stefan Wirtz, Florian Klämpfl

**Affiliations:** 1 Friedrich-Alexander-Universität Erlangen-Nürnberg, Institute of Photonic Technologies, 91052 Erlangen, Germany; 2 Department of Oral and Maxillofacial Surgery, University Hospital Erlangen, 91054 Erlangen, Germany; 3 Department of Medicine 1, University Hospital Erlangen, 91054 Erlangen, Germany; 4 Erlangen Graduate School in Advanced Optical Technologies, 91052 Erlangen, Germany

**Keywords:** Optics and photonics, Optical techniques, Optical imaging

## Abstract

Due to significant advantages, the trend in the field of medical technology is moving towards minimally or even non-invasive examination methods. In this respect, optical methods offer inherent benefits, as does diffuse reflectance imaging (DRI). The present study attempts to prove the suitability of DRI—when implemented alongside a suitable setup and data evaluation algorithm—to derive information from anatomically correctly scaled human capillaries (diameter: $10\,\upmu \hbox {m}$, length: $45\,\upmu \hbox {m}$) by conducting extensive Monte–Carlo simulations and by verifying the findings through laboratory experiments. As a result, the method of shifted position-diffuse reflectance imaging (SP-DRI) is established by which average signal modulations of up to 5% could be generated with an illumination wavelength of $\lambda =424\,\hbox {nm}$ and a core diameter of the illumination fiber of $50\,\upmu \hbox {m}$. No reference image is needed for this technique. The present study reveals that the diffuse reflectance data in combination with the SP-DRI normalization are suitable to localize human capillaries within turbid media.

## Introduction

Medical progress has been steadily advancing for numerous years, and new methods of diagnosing and treating diseases are constantly being developed. Due to relevant advantages such as the reduction of pain for the patient, the shortening of wound healing periods and the minimization of iatrogenic damages, invasive procedures are progressively replaced by minimally invasive or even non-invasive techniques wherever feasible.

In this context, non-invasive diagnostic techniques based on light are very promising. The most prominent approaches in this respect are probably pulse oximetry, used to monitor arterial oxygen saturation, and optical coherence tomography (OCT). Besides this, for example, the differentiation of cancerous and healthy tissue is possible by means of optical measures. What these techniques have in common is that they draw conclusions about the physiological state of the sample under investigation by exploiting its optical properties. In other words, it can be stated that an optimized determination of the optical properties of the tissue is fundamental for the development of new optical diagnostic modalities^1^.

One way of determining the optical properties of biological tissue is diffuse reflectance spectroscopy (DRS)^2^. In this method, the tissue is illuminated by a light source and the diffusely reflected light is spectroscopically analyzed. Usually, the light is guided using optical fibers. The detected spectrum contains encoded information about the optical parameters of the tissue which can be assessed by applying a corresponding model inversely^3^. In the literature, a large number of tissue types are reported to be investigateable by DRS^3^.

The depth from which the diffuse reflection (DR) information originates is derived from the optical penetration depth of light into skin. In the visible wavelength range, penetration depths between 100 and $1000\,\upmu \hbox {m}$ can be achieved^4,5^. Therefore, the information is primarily superficial. By selecting a certain illumination wavelength, it is possible to determine the tissue depth to be investigated^6–8^, which in turn equals the adjustment of the axial resolution of the system.

In the present study, emphasis will be placed on deducing conclusions about the capillary structure in human skin, i.e. in a turbid medium, from the diffuse reflection data. Medical methods such as capillaroscopy and cancer or shock detection employ this capillary structure to address medical questions^9^. Since diffuse reflectance imaging (DRI), which differs from DRS in that it uses only a single wavelength, is a cheap and easy to use method, it would be of great value for medicine and medical research if conclusions on the state of the capillary structure could be drawn using DRI.

The observation of the human capillary structure by means of DRS/DRI has already been reported in the literature—however, the dimensions of the capillary structures underlying these studies are significantly different from anatomically correct values^10,11^. The present study is therefore intended to investigate whether it is possible to detect capillary structures of realistic size and shape (diameter: $10\,\upmu \hbox {m}$, length: $50\,\upmu \hbox {m}$^12^, loop shape) employing our self-developed method of shifted position-diffuse reflectance imaging (SP-DRI), which is presented here for the first time. This question will be answered both by performing extensive Monte–Carlo (MC) simulations and by conducting laboratory experiments.

## Materials and methods

### Reconstruction of the capillary structure (SP-DRI normalization method)

For the reconstruction of the capillary structure from the simulative (details in “Simulation domain” section) and the experimental data (details in “Experimental domain” section), a novel normalization method was employed. This method has not yet been used in the literature in connection with DRI, therefore the expression SP-DRI (shifted position-diffuse reflectance imaging) will be defined in this publication. This algorithm is the central part of the present study.

This algorithm is the normalization of some diffuse reflectance (DR) data (matrix 1) by those DR data with slightly shifted *x* or *y* position of the light source at otherwise identical simulation/experimental parameters (matrix 2). For this method, the two matrices were shifted against each other in such a way that now the relative positions of the light source in both matrices were equal with respect to their *x* and *y* coordinates. This leads to a relative shift of the capillary structures to each other. Finally, one intensity matrix was divided pixelwise by the other one. The SP-DRI algorithm is extremely fast and takes only a few milliseconds to compute an image.

This procedure is borrowed from Raman spectroscopy, where it is known as shifted excitation raman difference spectroscopy (SERDS). In SERDS, the sample is excited with two adjacent wavelengths to solve the fluorescence background within Raman spectroscopy: The idea is that the fluorescence background is nearly identical in both spectra, while the characteristic Raman peaks are slightly shifted. By subtracting the two spectra, they become discernible^13^.

This idea (with the exception that in SERDS the two data sets are subtracted, while in SP-DRI due to more significant results a division is performed—the SP-DRI results oscillate around the value 1, as can be seen in “Results and preliminary discussion” section) is now being transferred to DRI by slightly shifting the excitation position. Thus, the signal variation does not occur in the wavelength range as in SERDS, but as a spatial shift: From a spatial point of view, the background noise remains almost unchanged, while the position of the signal is slightly offset.

Mathematically, this approach is equivalent to a computational edge filter, which is, due to the induced relative offset of the capillary structures to each other, sensitive to intensity changes generated by this capillary structure.

In the simulation domain, single images with a difference in the *y* value of the illumination fiber of $\Delta y=5\,\hbox {pixel}$ (px; small fiber diameter) or $\Delta y=11$ px (large fiber diameter) were used for this method (1 px $\widehat{=}~5\,\upmu \hbox {m}$). The illumination fiber shift was thus performed in parallel to the branches of the superficial vascular plexus. In the experimental domain, the fiber was moved by $\Delta x=55\,\upmu \hbox {m}$ towards a cantilever. SP-DRI method was followed by a filtering with a 2-D Gaussian smoothing kernel with a standard deviation of 2 px (simulation domain) and 5 px (experimental domain), respectively. The difference in the kernel size is subject to the different noise behavior in the two modalities investigated.

The generation of the raw data used in this study as input for the SP-DRI algorithm is described for both the simulation and the experimental domain in the following paragraphs.

### Simulation domain

#### Basic Monte–Carlo setup

The MC simulation was performed with MCXLAB V2019.4 on computers with MATLAB R2019a^14^. Two Nvidia Titan RTX (24GB of memory each) and two Nvidia Tesla P100 (16GB of memory each) were available for computation. The simulation volume used in this study is 500 $\times$ 500 $\times$ 500 pixels in size, with the length of one pixel set to $5\,\upmu \hbox {m}$. The illumination was implemented on the $-z$ boundary ($x=150$ px in all cases; $y=150$ px or $y=161$ px) as uniform cone beam in $+z$ direction with a defined half-angle; this constitutes the illumination of the system with a fiber with a core diameter $\varnothing _{\mathrm{Core}}=50\,\upmu \hbox {m}$ and a numerical aperture $A_{\mathrm{N}}=0.45$. The incident photon packets were detected on the entire $-z$ boundary and per detected packet its direction and coordinate of incidence at the boundary as well as the cummulative path lengths in each medium of its trajectory were stored.

With the exception of the surface upon which the illumination fiber and the detector were placed ($-z$ boundary), all boundaries of the simulated volume were absorbent in order to avoid undesired back reflection and to best represent realistic conditions. The $-z$ boundary took into account Fresnel reflection.

A photon packet was terminated when its weight was less than 1% of its initial weight to increase the simulation speed (to maintain the conservation of energy, the russian roulette approach was used). For each simulation run, $10^{11}$ photon packets were launched.

#### Simulated tissue model

Basically, the complexity of tissue models is almost arbitrarily scalable. One factor in this regard is the number of tissue layers considered—the higher this number, the more complex and thus more realistic is the tissue model. In the MC simulations of the present study, the stratum corneum is assumed to be the outermost skin layer, followed by an epidermal layer. The subsequent dermis consists of four layers and is flanked by subcutaneous tissue. Thus, the skin model used consists of a total of seven layers. Details on this can be found in Table 1.

**Table 1:** Skin layers considered in the MC simulations.

Tissue layer	Thickness along *z* axis ($\upmu \hbox {m}$)	Vasculature (diameter)
Stratum corneum	20	–
Epidermis	80	–
Papillary dermis	150	Loop ($10\,\upmu \hbox {m}$)
Upper blood net dermis	80	Horizontal cylinder ($30\,\upmu \hbox {m}$)
Reticular dermis	1500	–
Deep blood net dermis	80	–
Subcutaneous tissue	6000	–

**Figure 1:**
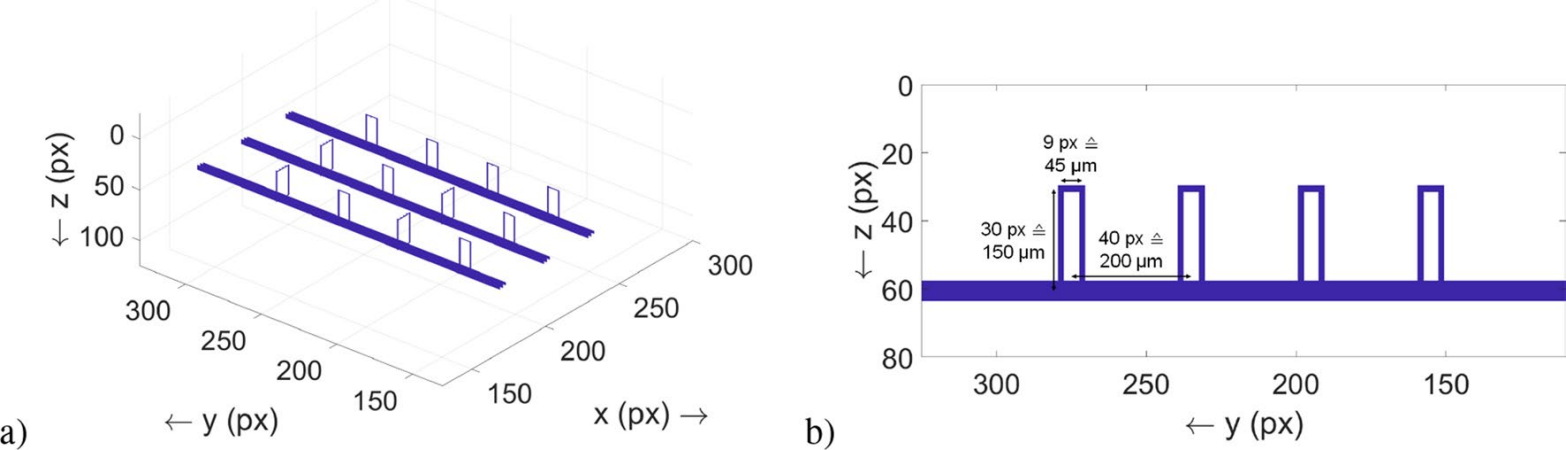
(**a**) Illustration of the vasculature considered in the MC simulation, consisting of the capillary loops and the superficial vascular plexus. Most of the capillary loops are aligned along the *y*–*z* plane, while four of them are aligned along the *x*–*z* plane. The coordinate system relates to the complete simulation volume. The spacing (along the *x* axis) between two branches of the superficial vascular plexus is 30 px ($\widehat{=}$ $150\,\upmu \hbox {m}$). (**b**) Detailed view of a vasculature branch with its dimensions. The dimensioning starts at the centers of the cylinders.

Another way to make the skin model more realistic and thus more complex is to take into account that haemoglobin is not evenly distributed in the tissue or tissue layer, but concentrated in blood vessels^15^. In the present study, haemoglobin is assumed to be concentrated in capillary loops and the superficial vascular plexus and can thus only be found in some of the tissue layers to be anatomically precise. Details on this vasculature can be obtained from Table 1 and Fig. 1, the dimensions are anatomically correct^12,16,17^.

#### Illumination wavelengths and optical properties

Since absorption and scattering of tissue are wavelength-dependent, the penetration depth of light into skin varies depending on the wavelength of the light. In the NIR and VIS regime photons of shorter wavelengths penetrate less deeply than those of longer wavelengths. As light with $\lambda =420\,\hbox {nm}$ is significantly backscattered within the epidermis and the superficial papillary dermis, it may not even reach the skin layers with cutaneous blood content. Light with $\lambda =585\,\hbox {nm}$, on the other hand, can penetrate into the reticular dermis and thus be absorbed by the blood present in the respective skin layers^7^. Thus, visible regime DRI of skin reports information that is weighted stronger towards the properties of the epidermis, while near-infrared DRI reveals information that is weighted stronger towards the properties of the dermis^4,6,8^.

The simulation is based on the wavelengths $\lambda =414\,\hbox {nm}$, $\lambda =424\,\hbox {nm}$ and $\lambda =540\,\hbox {nm}$. This takes into account the two wavelength regimes reported. In doing so, the respective wavelengths are chosen to meet the local maxima of the absorption curve of oxyhaemoglobin^18^. The selected illumination wavelengths thus allow to detect extremely small blood vessels: A high contrast can be achieved between the microvasculature filled with oxyhemoglobin and the surrounding tissue.

The four key parameters for characterizing and modelling the optical properties of tissue are the absorption coefficient $\mu _a$, the scattering coefficient $\mu _s$, the anisotropy factor *g* and the refractive index *n*. In order to create a realistic skin model, the single skin layers were also differentiated with regard to these optical parameters; the same applies to oxyhaemoglobin. In doing so, reference was made to numerical values and findings from corresponding literature sources. Table 2 contains the details and names the literature sources.

**Table 2:** Optical properties of the skin layers and the microvasculature considered in the MC simulation^18–22^.

Element	414 nm		424 nm		540 nm		*g*	*n*
	$\mu _a \,(\hbox {mm}^{-1})$	$\mu _s\,(\hbox {mm}^{-1})$	$\mu _a\,(\hbox {mm}^{-1})$	$\mu _s\,(\hbox {mm}^{-1})$	$\mu _a\,(\hbox {mm}^{-1})$	$\mu _s\,(\hbox {mm}^{-1})$		
Stratum corneum	1.57	50.00	1.46	50.00	1.00	50.00	0.90	1.53
Epidermis	3.44	14.60	3.19	13.96	1.81	7.84	0.85	1.34
Papillary dermis	0.86	13.50	0.80	13.36	0.45	11.09	0.80	1.40
Upper blood net dermis	1.23	13.50	1.14	13.36	0.65	11.09	0.90	1.39
Reticular dermis	0.74	13.50	0.68	13.36	0.39	11.09	0.76	1.40
Deep blood net dermis	1.47	13.50	1.37	13.36	0.78	11.09	0.95	1.39
Subcutaneous tissue	0.69	7.00	0.64	7.00	0.36	7.00	0.80	1.44
Microvasculature	283.00	8.00	203.17	8.00	28.75	8.00	0.96	1.36

#### Post-processing of detector data

The raw data of the simulation provides information on how many photon packets were registered at each position of the $-z$ boundary. Of interest, however, is the diffuse reflection in terms of an intensity distribution on the $-z$ boundary. During post-processing, the weight of the photon packets at the $-z$ boundary (the packets are attenuated along their trajectories) is calculated using Beer’s law.

The fact that diffuse reflection is often captured using a fiber bundle is also taken into account in post-processing. In this paper, a numerical aperture $A_{\mathrm{N}}=0.66$ was assumed for the detection fibers. For the simulation domain, this post-processed data was used for the reconstruction of the vasculature structure as it is explained in “Reconstruction of the capillary structure (SP-DRI normalization method)” section.

### Experimental domain

**Figure 2:**
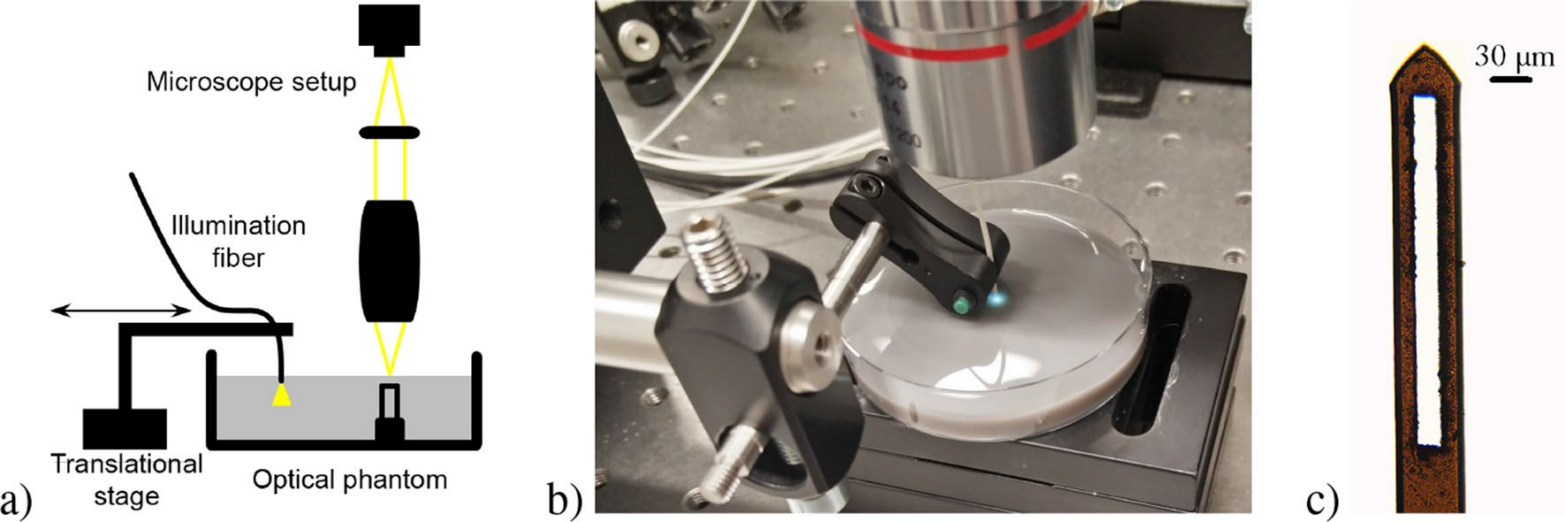
(**a**) Schematic of the experimental setup consisting of an optical phantom with a cantilever incorporated to imitate a capillary loop, a traversable illumination by means of a glass fiber and a microscope setup to image the diffuse reflection from the phantom surface on a camera chip. By way of illustration, the cantilever is turned by 90 degree in the sketch. (**b**) Photograph of the setup. (**c**) Transmitted light microscope image of the cantilever used in the optical phantom. To optimally mimic a capillary loop, material was removed from the cantilever over a large area by ultrashort pulse laser ablation.

The laboratory experiments aim to validate the results of the MC simulation in a practical manner. Therefore, they have to be regarded as a proof of concept. Basically, the setup consists of three main parts. A sketch and a photograph of the experimental set-up are shown in Fig. 2a,b, respectively.

First, the optical phantom with a matrix composed of distilled water, lipid emulsion (Fresenius Kabi, Intralipid 20%) and black ink (Pelikan, Fount India). Their volumetric proportions were chosen such that the optical properties of the phantom are similar to those of skin in terms of $\mu _a$, $\mu _s$, *g* and *n* at $\lambda \approx 540\,\hbox {nm}$, which was used for illumination. Since the phantom used in the experiments is a single-layered one, the target values for the optical properties were determined by averaging the respective values of the epidermis and the papillary dermis. According to literature references^23^ and own spectrophotometric measurements (Shimadzu, UV-3600), this could be achieved with a mixture of 20 parts distilled water, 1 part lipid emulsion and 0.02 parts ink.

An uncoated tipless cantilever (Nanosensors, TL-CONT; length: $450\,\upmu \hbox {m}$, width: $50\,\upmu \hbox {m}$, thickness: $2\,\upmu \hbox {m}$; material: silicon) from atomic force microscopy (AFM) was adopted as the structure inside the phantom. Using ultrashort pulse laser ablation, the cantilever was processed to optimally mimic a capillary loop: It was possible to generate bars with a width of $13\,\upmu \hbox {m}$, while the overall width of the cantilever and its arc shape are inherently consistent with the human anatomical dimensions. A microscope image of the processed cantilever is shown in Fig. 2c.

Although the cantilever is made of silicon and therefore reflective in contrast to the absorbing vasculature structures in the simulation model, this does not affect the proof of concept: In both cases, an opaque disturbance structure is introduced. The filling level of the phantom compound was chosen such that the phantom surface was $150\,\upmu \hbox {m}$ above the top of the cantilever—this corresponds to the conditions of the simulation domain and is anatomically correct.

Second, there is the illumination, which was realized by means of a glass fiber ($\varnothing _{\mathrm{Core}}=50\,\upmu \hbox {m}$) with the light of a mercury-vapor lamp coupled into it. To prevent the image information from being blurred, the spectral range of the light source was narrowed by means of a long pass ($\lambda _{\mathrm{Cut-On}}=500\,\hbox {nm}$) and a short pass filter ($\lambda _{\mathrm{Cut-Off}}=650\,\hbox {nm}$) so that the illumination spectrum was finally dominated by the strong intensity peak of the mercury-vapor lamp at $\lambda \approx 540\,\hbox {nm}$ and the weaker peak close by at $\lambda \approx 570\,\hbox {nm}$.

The glass fiber could be moved parallel to the phantom surface with micrometre precision by means of a motorized translational stage. Perpendicular to the phantom surface, the glass fiber was positioned in such a way that its tip was just completely immersed in the liquid to minimize specular reflection.

The diffuse reflection, thirdly, was detected with a microscope setup consisting of an infinity corrected objective (Mitutoyo, Plan Apo, $f=40\,\hbox {mm}$, $A_{\mathrm{N}}=0.14$) and a plano-convex lens ($f=150\,\hbox {mm}$). With this, the phantom surface located above the cantilever was imaged onto a monochrome CMOS camera (Flir, BFS-U3-122S6M-C) with a magnification factor of $M=3.75$.

For data generation, images were taken with the exposure time of the camera set to $300\,\hbox {ms}$ and the color depth set to 12 bit. All ambient light sources were turned off. With the motorized translational stage, during data generation the lateral position of the illumination fiber relative to the surface of the optical phantom could be changed to collect all images necessary for the reconstruction of the vasculature structure as it is explained in “Reconstruction of the capillary structure (SP-DRI normalization method)” section.

## Results and preliminary discussion

For the simulative part of the investigation, in the following the results for the illumination wavelengths in the range of $\lambda =420\,\hbox {nm}$ are shown. For the illumination wavelength $\lambda =540\,\hbox {nm}$, equivalent results were obtained, although with slightly weaker modulations.

### Functionality of SP-DRI for the detection of the capillary structure (simulation domain)

**Figure 3:**
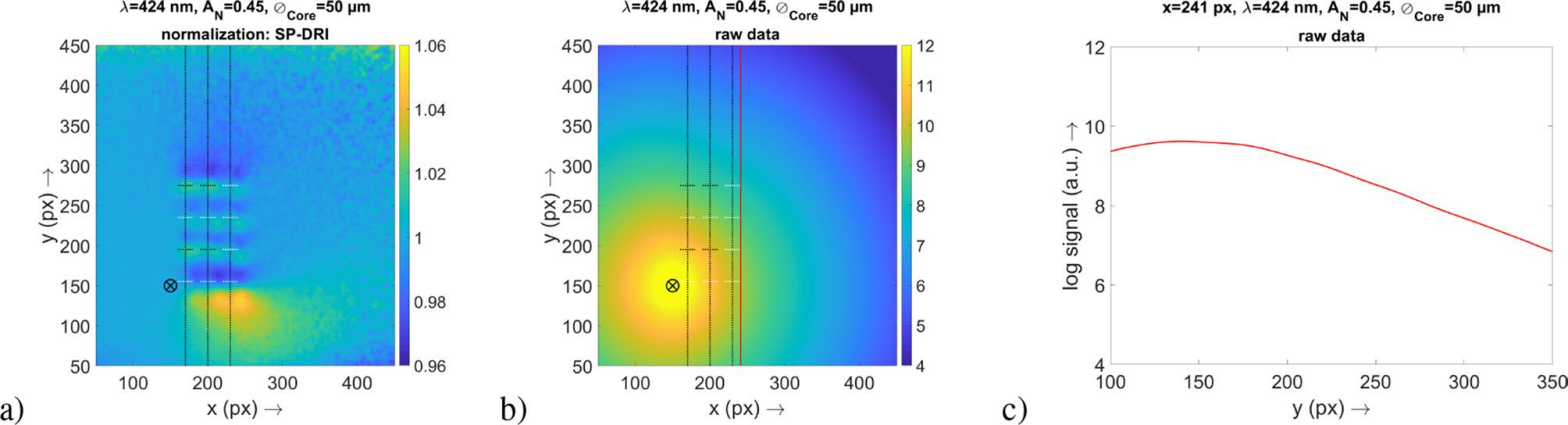
(**a**) Result for the SP-DRI method. The default vasculature is encountered and the positions of the vasculature and the capillary loops are indicated by the dashed lines (the capillary loops that are actually aligned along the *y* axis are marked in white, compare Fig. 1a. The position of the illumination fiber is also shown. (**b**) Logarithmic signal for the raw data set 1 which was used for the SP-DRI normalization in (**a**). The position of the cross-sectional plane is also shown. (**c**) Graph of the cross-sectional plane at $x=241$ px for the raw data in (**b**).

The results of the *x*–*y* maps in Fig. 3a clearly show that the vasculature can be identified at a glance. Furthermore, the respective signals are located at positions where the capillary loops are localized according to the structure of the simulation volume. There is a clear difference between this data and the raw data also shown in Fig. 3—here, the capillary structure is not visible.

Due to the division of the two DR data sets upon which the SP-DRI method is based (compare “Reconstruction of the capillary structure (SP-DRI normalization method)” section), it is evident that a capillary loop must always be located at a position where the SP-DRI signal passes through the value 1. Based on what has been defined in this study as data set 1 and data set 2, respectively, in the following results section a capillary loop is always present when the SP-DRI signal drops from a value above 1 (yellow or green, respectively) to a value below 1 (dark blue).

Two important findings are evident when taking a look at Fig. 3: First, the data of the SP-DRI normalization are not superimposed by the signal of the illumination fiber. Secondly, the signal contains only information of the capillary loops, but not of the slightly deeper superficial vascular plexus.

Due to scattering and absorption by the capillaries, also the (structure-free) areas behind the capillaries are altered regarding their intensities, leading to a blur in this regions. With the SP-DRI normalization, this blur is present in both images required for this method so that it factually does not take effect. Since the superficial vascular plexus also emerges only blurred due to its depth, the same applies to it. As a result, only the capillary loops become visible with this normalization method.

### Specificity of SP-DRI in detecting the capillary structure (simulation domain)

To prove the specificity of the SP-DRI method as a tool to facilitate the detection of the capillary structure, parts of the vasculature were removed from the simulation volume and it was examined whether the SP-DRI signal disappears at the corresponding position. In one setup, the capillary loops were removed from all three vasculature branches at $y=275$ px (thus, in comparison to the default setup, each branch had only three capillary loops instead of four), in another setup one entire vasculature branch was removed at $x=230$ px (thus, in comparison to the default setup, there were only two vasculature branches left, but each of these branches still had four capillary loops).

**Figure 4:**
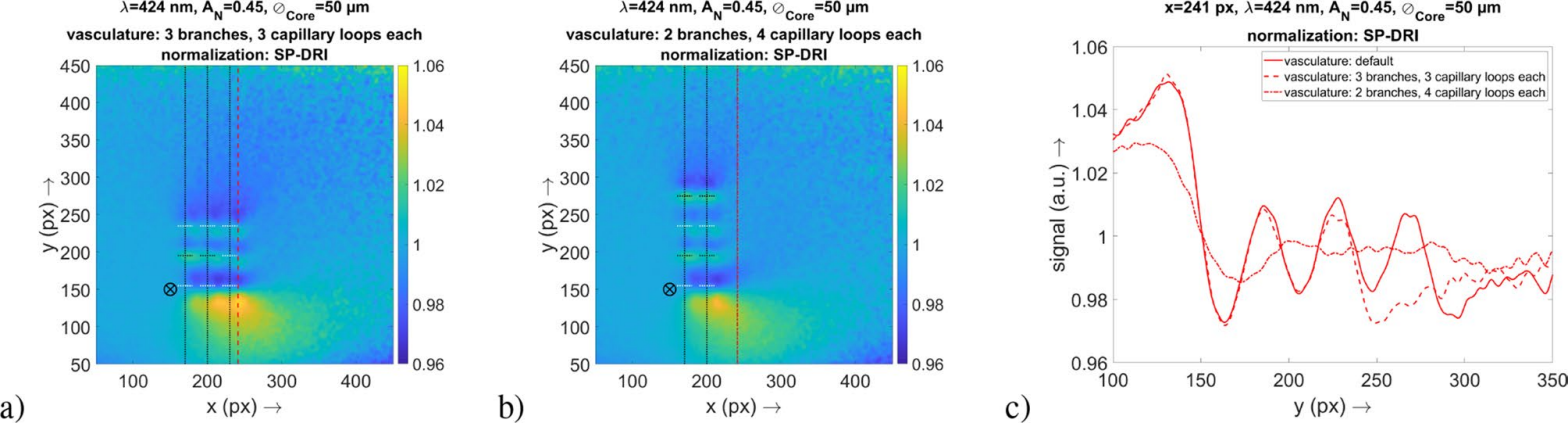
Results for the examination of the specificity with a difference in the number of capillary loops and vasculature branches. The positions of the vasculature and the capillary loops are indicated by the dashed lines (the capillary loops that are actually aligned along the *y* axis are marked in white). The position of the illumination fiber (size not according to scale) and of the cross-sectional plane is also shown. (**a**) SP-DRI signal for the setup with only three capillary loops at each of the vasculature branches. (**b**) SP-DRI signal for the setup with only two vasculature branches. (**c**) Graph of the cross-sectional plane at $x=241$ px to compare the two validation setups with the default setup as shown in Fig. 3.

The positions of the capillary loops can be identified already by simply taking a look at the signals in the *x*–*y* map. It is important to note that regions between two adjacent vasculature branches can be identified as structureless. Since the spatial arrangement of the capillary loops relative to each other is in an anatomically correct scale in the simulation volume, this observation permits to conclude that the SP-DRI method has a sufficient lateral resolution to detect capillaries in human skin.

For further illustration of these results, a graph with the cross-sectional plane at $x=241$ px is presented in Fig. 4, which again shows the two effects of importance: The comparison of the signal from a vascular branch with three capillary loops with the signal from a vascular branch with four capillary loops (default setup) shows that the corresponding peak at position $y\approx 275$ px has disappeared. The same is true for the second described setup: The comparison of the signal from the setup with two vascular branches with the signal from the setup with three vascular branches (default setup, again) clearly indicates the absence of the corresponding capillary loops.

The evidence in this section therefore suggests a high specificity of the SP-DRI method: The absence of a capillary loop is reflected as such by the technique. In addition, the slightly deeper superficial vascular plexus is not falsely detected as a capillary loop. With SP-DRI it is therefore possible to explicitly derive information on the capillary loops.

In the context of Fig. 4, another strength of the SP-DRI method should be emphasized at this point: The comparison of signals from around $x=180$ px and $x=210$ px with those from around $x=240$ px shows that the orientation of a capillary loop has no influence on its detectability—there are no differences in the detected signals, even though the capillary loops are partly aligned along the *x* axis and partly along the *y* axis. Since in human skin the capillary loops are not oriented in an orderly fashion, this property of the SP-DRI method is advantageous for a future in vivo application.

### Two-point discrimination of SP-DRI for the detection of the capillary structure (simulation domain)

To demonstrate the ability of two-point discrimination of the SP-DRI normalization as a tool to facilitate the detection of the capillary structure, the SP-DRI method should be validated by randomly placing two vasculature branches each with two capillary loops (two of them aligned along the *x* axis, two along the *y* axis) in the simulation volume. The depth *z* of the vasculature was defined as in the default setup, see Fig. 1.

**Figure 5:**
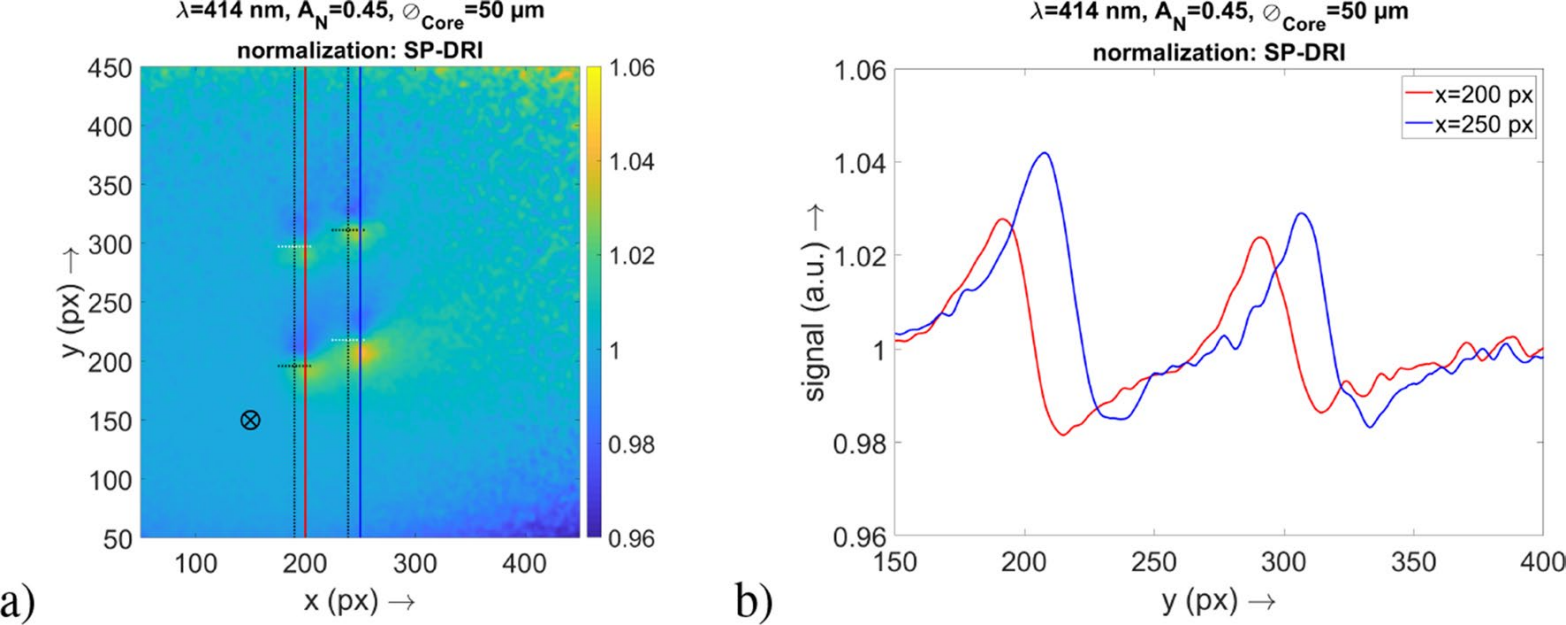
Results for the examination of the ability of two-point discrimination with a random distribution of the vasculature. (**a**) Map with the positions of the four capillary loops indicated by the dashed lines. The capillary loops that are actually aligned along the *y* axis are marked in white. The positions of the illumination fiber and of the cross-sectional planes are also shown. (**b**) Cross-section at the two relevant *x* positions.

Figure 5 shows the corresponding SP-DRI signals. It is evident from the plot with the SP-DRI signal of the detector area that an easy and clear determination of the positions of the capillary loops is possible for the random vasculature configuration. This statement is also supported by the diagrams containing the corresponding cross-sectional planes.

It is important to note once more that the data doesn’t contain information about the connective superficial vascular plexus that is located slightly deeper within the tissue volume. This is true regardless of the orientation of the single capillary loops, as the data shows.

The evidence in this section therefore suggests a sufficient ability of two-point discrimination of the SP-DRI method: If a capillary loop is present, it can be identified and detected as such by the technique.

### Proof of concept (experimental domain)

**Figure 6:**
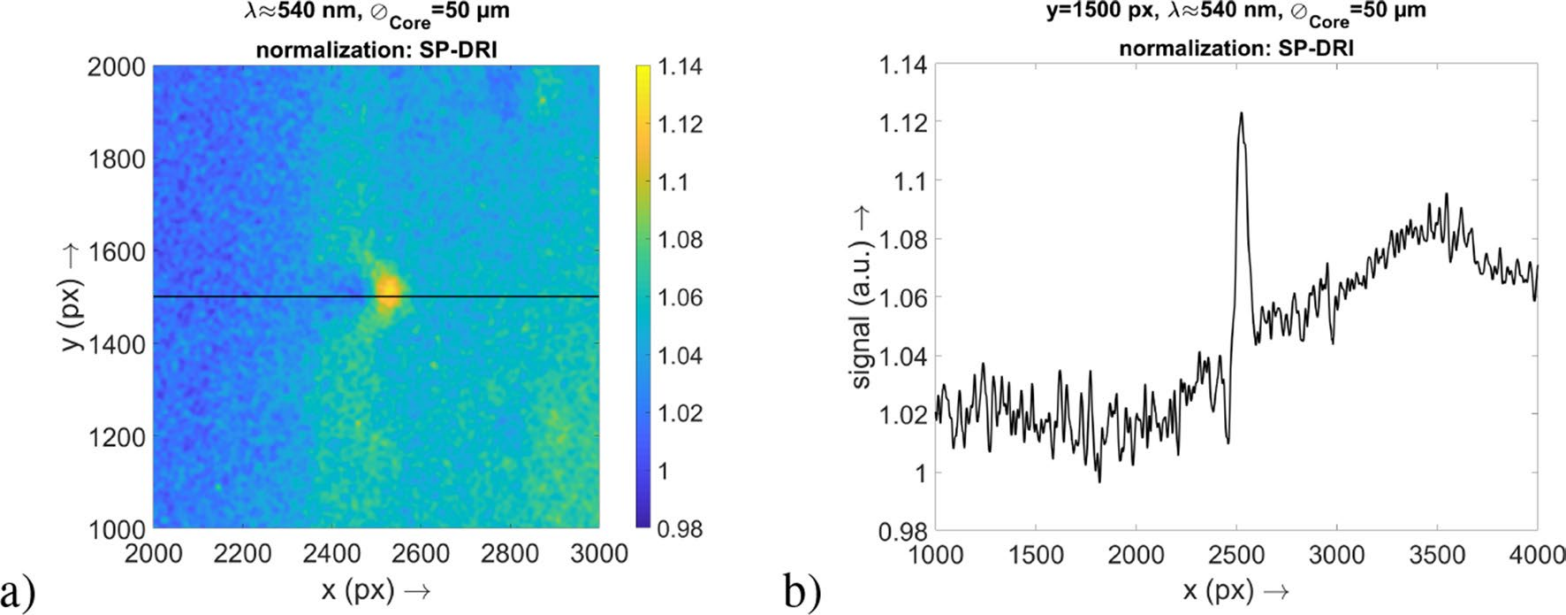
Results of the laboratory experiment for the proof of concept of the SP-DRI normalization method. (**a**) Image of the result, where the position of the cantilever structure inside the optical phantom is clearly visible. The position of the illumination fiber is not shown here as it is outside of the range illustrated on the map. (**b**) A cross-section through the image from (**a**) at the position $y=1500$ px.

In order to investigate whether the SP-DRI normalization method is also in practice suitable for the detection of perturbations on the scale of human capillaries, the laboratory experiment already described was carried out. The associated results are summarized in Fig. 6.

It is evident that the cantilever structure can be clearly identified as such in the *x*–*y* map and the cross-section through the perturbation along the *x* axis. Since this structure is of the order of a capillary loop, it is reasonable to assume that the latter can be detected with the SP-DRI normalization method, in particular also because the matrix of the employed optical phantom adequately mimics the optical properties of skin.

The width of the structure as reflected in the diffuse reflection data is in line with expectations: By taking into account the width of the cantilever, the magnification factor of the detection setup and the size of a camera pixel, this cantilever width corresponds to a value of 60 px. Furthermore, the distortion in the SP-DRI signal is consistent with the location of the cantilever relative to the camera image.

In this respect, with an AFM cantilever it has been successful to introduce a structure that is stable and practical to handle despite its very small dimensions. It was possible to transfer this already small structure into the shape of a capillary loop by laser processing, with the loop having anatomically correct proportions. This approach allowed to perform reproducible experiments as it would have been almost impossible with, for example, harvested porcine capillaries as they are neither stable nor robust.

Compared to the absorbing human capillary loop, this cantilever is highly reflective. This, however, does not alter the experiments at all: The reflecting silica increases the backscattering, resulting in a brighter area instead of a darker one at the surface. This effect will create a photon source instead of a sink relative to the substrate. Accordingly, in the simulation domain with its absorbing structures a strong positive deflection in the SP-DRI signal is followed by a small negative one. In contrast, in the experimental domain with its reflecting structure a small negative deflection is followed by a strong positive one. The two configurations thus produce comparable SP-DRI signals that only differ in the sequence of the peaks, while their amplitudes are virtually equal. In terms of a mathematical differentiation (which the SP-DRI method actually is), this behavior is in line with expectations. In summary, the cantilever as interfering structure serves as a valid proof of concept: SP-DRI is capable of detecting pertubations of the order of human capillaries—absorption and reflection, respectively, are indicated by the sign of the signal.

## General discussion

The progress of minimally and non-invasive medical techniques is driven not least by optical technologies with their inherent advantages^1^. One such method is diffuse reflectance imaging, which has already proven its suitability for medical imaging^2^—however, until now only relatively large structures have been imaged^24,25^. The present study has now been able to clearly show that DRI is also capable of detecting very small patterns: Using appropriate data normalization routines, human capillaries with their diameter of $10\,\upmu \hbox {m}$ can be detected from diffuse reflection data. In this study, the normalization method of shifted position-diffuse reflectance imaging (SP-DRI) was contrived and investigated in detail for this purpose. This method was adapted to DRI from Raman spectroscopy, where it is known as shifted excitation raman difference spectroscopy (SERDS)^26^.

The SP-DRI normalization proved to be remarkably successful: The capillary structure could be made clearly visible and its localization also corresponded to the positions derived from the simulation model. Since the goal of the method is to detect spatial structures and (at a later stage of research) their alterations over the course of time (e.g. dilatation or constriction) and not, as otherwise common in DRI, to extract optical properties from biological tissues, no time- and resource-intensive inverse algorithm is involved—data acquisition is thus possible in almost real time.

SP-DRI is quite easy to implement in practice: It only requires two illumination fibers (or one movable) placed directly next to each other, an alternating illumination of the tissue site and an approach for capturing the diffuse reflection—this can be done either directly with a microscope imaging approach or by extracting the diffuse reflection using detection fibers or by means of a CCD/CMOS chip (with suitably small pixels) placed directly on the surface of interest. If SP-DRI is implemented using the latter of the three approaches, a particular constructional strength of this method becomes apparent: The setup can be kept extremely small and no expensive optical components are required, not even lenses. This allows, on the one hand, a very cost-effective design and, on the other hand, no complex alignment of the setup is necessary. Due to the short exposure and calculation times, acquisition and processing are very robust against motion artifacts.

Not least with respect to the aspects mentioned in the previous two paragraphs, the SP-DRI method differs from methods such as ptychography, for example, that require substantially more effort in their practical implementation (alignment, microscopy setup), are limited in their choice of illumination (coherent light source) and demand a complex data processing (phase retrieval). A very similar argumentation applies to techniques such as optical coherence tomography (OCT) or its use in angiography (OCT-A)^27,28^, respectively, photoacoustic tomography (PAT)^29–31^ and diffuse optical tomography (DOT)^32,33^. Other approaches reported in the literature, such as that of Bish et al., also suffer from these disadvantages, and in addition, the attempt of these authors can only achieve a resolution of $100\,\upmu \hbox {m}$^34^.

Due to its inherent advantages, in the present paper a comprehensive validation of the SP-DRI method for the detection of the capillary structure was conducted both in simulation and experimental domain. In this regard, it was investigated whether the lateral resolution of the SP-DRI technique is sufficient for this task. The single capillary loops were placed in anatomically correct spacing to each other in the simulation volume. Under these conditions, the different capillary loops were clearly distinguishable in the SP-DRI signal. As only 25% to 50% of the human capillary loops are perfused with blood at any given time^16^, this is a very conservative estimation (if only a fraction of all capillary loops is perfused at the same time, this numerically corresponds to a larger lateral spacing—therefore, the required lateral resolution of the DRI system could even be lower).

In order to also illustrate the scenario that not all capillary loops of a vasculature branch are supplied with blood at the same time, four capillary loops (i.e. less than in the fully perfused default simulation setup) were randomly arranged in the simulation volume. The detection of the capillary loops was still possible with appropriately high signals.

In the sense of a validity check, the random capillary distribution setup just described represents the testing of the ability of two-point discrimination: May a capillary loop that is present in the simulation volume be identified and detected as such by the SP-DRI technique? According to the results, the method is sensitive to the detection of capillary loops—which is true regardless of the orientation of the single capillary loop within the simulation volume.

In addition to that, specificity is of importance: It was shown that the absence of a capillary loop is reflected as such by the SP-DRI technique. This may sound rather trivial, but points to an important property of SP-DRI (when it is used in connection with the optimal parameters described before): The slightly deeper superficial vascular plexus is not falsely detected as a capillary loop by the technique. It is therefore possible to detect exclusively the position of the capillary loops with the SP-DRI method.

In this study, the detection of capillary loops was demonstrated with illumination wavelengths in the range of $\lambda =420\,\hbox {nm}$. Although this is a wavelength range that is preferably avoided in the context of medical diagnostics due to the comparatively high energy of the photons, it must be pointed out that only low doses of incoherent light are required for DRI and that the diagnose system will only be applied for a short time. Therefore, it can be assumed that the threshold for the exposure of skin can be maintained^35^ and that this does not impose a limitation on the applicability of the system; detailed calculations on this will have to follow as soon as a first properly dimensioned system for the in vivo application is available. The authors also tested the SP-DRI method with an illumination wavelengths of $\lambda =540\,\hbox {nm}$. Even with this configuration, the detection of the capillary loops within turbid media was possible, although the achievable modulation was a bit lower due to the reduced contrast between hemoglobin and the surrounding tissue. For this reason, in this article selected wavelengths in the range of $\lambda =420\,\hbox {nm}$ were shown.

It is important to note at this point that the SP-DRI method is still functional for the detection of the capillary structure even if the shift of the illumination fiber is perpendicular to the branches of the superficial vascular plexus (and not parallel to it as presented in the results section of this paper): This scenario has also been simulated by the authors; again, the capillary loops were clearly distinguishable from the superficial vascular plexus.

The method also proved to be functional in a practical laboratory experiment. The cantilever (from an AFM) used and modified in the sense of a capillary loop could be identified as such in the SP-DRI data, with the width and position of the structure represented in the data being in good agreement with the actual position within the optical phantom.

In summary, with the SP-DRI method a technique has been developed to easily obtain information from DR data about the position of structures of the order of human capillaries within a turbid medium.

## Conclusion

For the first time, the present study was able to show that it is feasible to detect anatomically correctly scaled human capillaries using DRI alongside a suitable setup and data evaluation algorithm. It is essential to emphasize that this was possible with only one single wavelength and a core diameter $\varnothing _{\mathrm{Core}}=50\,\upmu \hbox {m}$ of the illumination fiber. The normalization method of SP-DRI (shifted position-diffuse reflectance imaging), which enabled this imaging and which was applied for the first time in this study in the DRI context, proved to be a suitable technique to obtain the corresponding information from the detector raw data. By choosing an illumination wavelength in the range of $\lambda =420\,\hbox {nm}$, average signal modulations of 5 percent originating from capillary loops with a diameter of only $10\,\upmu \hbox {m}$ could be generated—in the experimental domain, this value was even about three percent higher.

Based on the available simulation data, the SP-DRI normalization was validated for the detection of the capillary structure and both its specificity and the ability of two-point discrimination could be confirmed. Not least, a random arrangement of the vasculature in the simulation volume and the successful detection of the capillary loops in this very setup by an investigator who had no knowledge of the positions of the randomly arranged structures proved the strength of the method. Beyond that, the SP-DRI normalization could already be practically examined in a laboratory experiment. This allowed the simulation findings and thus the strength of the method to be confirmed.

As a next step, the method is now to be validated on human tissue. Furthermore, an approach is to be developed that automatically recognizes the position of the capillary loops from the SP-DRI data, especially in very large data sets—in this respect, the use of neural networks seems to be particularly promising. Furthermore, due to the excellent performance of the SP-DRI method for the detection of capillary structures, it is crucial to make this method also available for the detection of other microscopically small structures: This requires a broader generalization of the discussed principle and it is necessary to clarify what the ultimate spatial resolution and imaging depth in a medium with given optical properties are.
